# Prevalence and Predictors of Antibiotic Administration during Pregnancy and Birth

**DOI:** 10.1371/journal.pone.0082932

**Published:** 2013-12-10

**Authors:** Jakob Stokholm, Susanne Schjørring, Louise Pedersen, Anne Louise Bischoff, Nilofar Følsgaard, Charlotte G. Carson, Bo L. K. Chawes, Klaus Bønnelykke, Anne Mølgaard, Karen A. Krogfelt, Hans Bisgaard

**Affiliations:** 1 Copenhagen Prospective Studies on Asthma in Childhood, Health Sciences, University of Copenhagen, Naestved Hospital, Naestved, Denmark; 2 Copenhagen Prospective Studies on Asthma in Childhood, Health Sciences, University of Copenhagen, Copenhagen University Hospital, Gentofte, Copenhagen, Denmark; 3 Department of Microbiological Surveillance and Research, Statens Serum Institut, Copenhagen, Denmark; UCL Institute of Child Health, University College London, United Kingdom

## Abstract

**Background:**

Antibiotic treatment during pregnancy and birth is very common. In this study, we describe the estimated prevalence of antibiotic administration during pregnancy and birth in the COPSAC_2010_ pregnancy cohort, and analyze dependence on social and lifestyle-related factors.

**Methods:**

706 pregnant women from the novel unselected Copenhagen Prospective Study on Asthma in Childhood (COPSAC_2010_) pregnancy cohort participated in this analysis. Detailed information on oral antibiotic prescriptions during pregnancy filled at the pharmacy was obtained and verified longitudinally. Information on intrapartum antibiotics, social, and lifestyle-factors was obtained by personal interviews.

**Results:**

The prevalence of antibiotic use was 37% during pregnancy and 33% intrapartum. Lower maternal age at birth; adjusted odds ratio (aOR) 0.94, 95% CI, [0.90-0.98], *p* = 0.003 and maternal smoking; aOR 1.97, 95% CI, [1.07-3.63], *p* = 0.030 were associated with use of antibiotics for urinary tract infection during pregnancy. Maternal educational level (low vs. high), aOR 2.32, 95% CI, [1.24-4.35], *p* = 0.011, maternal asthma; aOR 1.99, 95% CI, [1.33-2.98], *p* < 0.001 and previous childbirth; aOR 1.80, 95% CI, [1.21-2.66], *p* = 0.004 were associated with use of antibiotics for respiratory tract infection during pregnancy. Lower gestational age; aOR 0.72, 95% CI, [0.61-0.85], *p* < 0.001, maternal smoking; aOR 2.84, 95% CI, [1.33-6.06], *p* = 0.007, and nulliparity; aOR 1.79, 95% CI, [1.06-3.02], *p* = 0.030 were associated with administration of intrapartum antibiotics in women giving birth vaginally.

**Conclusion:**

Antibiotic administration during pregnancy and birth may be influenced by social and lifestyle-factors. Understanding such risk factors may guide preventive strategies in order to avoid unnecessary use of antibiotics.

## Introduction

Across various cultural and healthcare settings, antibiotics are among the most widely used drugs in pregnancy[1]. In westernized societies prescription rates differ substantially with 20% - 49% of women being treated with antibiotics during pregnancy[2–8]. Prescribing drugs during pregnancy presents a challenge to the physician; infections need to be treated, while protecting the fetus against possible side-effects from the drugs[9].

An increase in intrapartum antibiotics has been reported over the past decade as a result of prenatal screening for Group B Streptococcus (GBS)[10,11]. Intrapartum antibiotic prophylaxis is recommended for GBS positive women[12]. This screening procedure is not applied in Denmark. Instead prophylaxis is initiated upon a non-culture based risk factor approach[13]. Postpartum infections in the mother can be reduced after caesarian section when prophylactic antibiotics are administered during the procedure[14]. 

A few register based studies have been conducted with a focus on predictors for antibiotic treatment in pregnancy[3,4]. The rate of antibiotic usage seems influenced by both social and lifestyle-factors. Previous antibiotic administration in pregnancy, frequent visits to the physicians and maternal asthma status increases the usage[4]. The antibiotic administration rate during pregnancy varies according to the age of the woman, the place of residence and the social status of the woman. A higher antibiotic administration has been described in women who were welfare recipients, unemployed or pensioners[3].

The objective of this study was to analyze the prevalence of antibiotics administered during pregnancy and birth in the COPSAC_2010_ pregnancy cohort, and to study factors affecting this usage. We hypothesize that social and lifestyle-related factors may drive the prescription pattern. 

## Methods

### Ethics

The study was performed according to the principles of the Declaration of Helsinki and was approved by the Ethics Committee of Copenhagen (H-B-2008-093) and the Danish Data Protection Agency (2008-41-2599), and written informed consent was obtained from all families. 

The study is reported in accordance with the STROBE guidelines[15]. 

### Study population

The Copenhagen Prospective Study on Asthma in Childhood 2010 (COPSAC_2010_) is an ongoing Danish cohort study of 738 unselected pregnant women and their children followed prospectively from pregnancy week 24 in a protocol previously described in details [16] and designed from the first COPSAC birth cohort (COPSAC_2000_)[17–19]. Exclusion criteria were chronic cardiac, endocrinological, nephrological or lung disease other than asthma. Data validation and quality control followed the guidelines for good clinical practice. Data was collected during visits to the clinical research unit and stored into a dedicated online database. 

### Information on antibiotic use

Detailed information on antibiotic usage was obtained during interviews with the participants at the COPSAC research clinic at weeks 24 and 36 of gestation, and 1 week postpartum. This information was validated with the mother 1 week postpartum by data from The Danish Medical Agency’s Register, which includes records on all drugs filled at the pharmacy. Prescriptions filled at the pharmacy are linked with a unique person identification number. This double check procedure eliminated recall bias and excluded antibiotics collected at the pharmacy but not ingested by the participants. Treatments administered in hospitals or abroad were missing in the registers but obtained through the interviews. The combined information was stored for each participant. Information on intrapartum antibiotics was obtained by interviews at the COPSAC research clinic 1 week postpartum. If the mother did not know, the birth journal was inspected. All women giving birth by caesarian section were treated with prophylactic intrapartum antibiotics. 

Oral antibiotic usage during pregnancy was in the prevalence description analyzed both as a dichotomized (yes/no) and as a categorized variable by most likely treatment indication (A: Urinary tract infection (UTI) antibiotics (J01CA08, J01EBxx, J01XExx); B: Respiratory tract infection (RTI) antibiotics (J01CAxx excl. J01CA08, J01CExx, J01FAxx); C: other antibiotics (J01CFxx, D06BXxx, J01AAxx, P01ABxx). In the predictor analysis, the group C (other antibiotics) was removed due to low numbers. Women who received both UTI and RTI treatment in pregnancy were examined as cases in both of the predictor analyses. The control groups were defined as all the remaining women. Prevalence analyses were performed in each trimester of pregnancy (first (≤14 weeks of gestation), second (>14 - ≤26 weeks of gestation), and third (>26 weeks of gestation)). Number of treatments were in the prevalence description analyzed as a numeric variable and in the risk analysis as a dichotomized (multiple treatments yes/no) variable. Intrapartum antibiotics were analyzed only as a dichotomized (yes/no) variable. As all women giving birth by caesarian section were treated with intrapartum antibiotics, we only analyzed predictors for antibiotic administration during vaginal birth. 

### Lifestyle and social factors (Covariates)

Information on maternal age at birth, gestational age (used only in the analysis of intrapartum antibiotics), race (Caucasian/non-Caucasian), parity, number of older children at home, maternal asthma status (doctor diagnosed asthma), alcohol intake (> 1 unit/week), smoking at any time-point during pregnancy, maternal educational level (low; elementary school or college graduate, medium; medium length or tradesman, high; university candidate) and household income (low; below 50.000 Euro, medium; 50.000 - 110.000 Euro, high; above 110.000 Euro) during pregnancy was obtained during the scheduled clinical visits at gestational week 24 and 36, and 1 week postpartum. 

### Statistical analysis

Chi-square test, student’s t-test, or Wilcoxon rank-sum test was used for analyzing simple associations between social and lifestyle-related variables and antibiotic administration during pregnancy and birth in the baseline characteristics. Chi-square test was used for dichotomized and categorical variables and student’s t-test for continuous variables. Wilcoxon rank-sum test was used for the non-parametric value older children at home. The ordered categorical variables: household income and maternal education were further analyzed using a univariate Cochran-Armitage Trend Test. Significant associations were analyzed further by multiple logistic regression including all covariates in the model using backward selection with p < 0.10. Adjusted estimates were expressed as odds ratios with corresponding 95% confidence intervals. A significance level of 0.05 was used in all analyses. Missing data was treated as missing observations. The data processing was conducted using SAS version 9.3 for Windows (SAS Institute Inc, Cary, NC, USA). 

## Results

### Prevalence of antibiotic usage

Complete data on oral antibiotic administration during pregnancy was available for 706 women from the COPSAC_2010_ pregnancy cohort of 738 women (96%). Of the 706 pregnant women 260 (37%) had received oral antibacterial therapy on one or several occasions during pregnancy, a total of 433 treatments. The most prevalent administered antibacterial agents during pregnancy were UTI antibiotics (21% of the women) and RTI antibiotics (21% of the women). Only 1% received other antibacterial drugs. A single treatment during pregnancy was most common (24% of the women), 7% of the women received 2 treatments, 3% received 3 treatments, 2% received 4 treatments, and 1% received 5 treatments or more. Treatment with antibiotics was least common in the first trimester of pregnancy in which 13% of the women received treatment, followed by the second trimester in which 16% of the women received treatment. The highest treatment prevalence was observed in the third trimester with 18% of the women treated. (Table 1) Information on intrapartum antibiotics was available in 704 women (95%). Of the 704 pregnant women 229 (33%) received intrapartum antibiotics. Among these, 157 women (69%) gave birth by caesarian section and all of these received intrapartum antibiotics. Among the 547 women giving birth vaginally, 72 (13%) received intrapartum antibiotics.

**Table 1:** Prevalence of antibiotic administration during pregnancy, 706 women.

**Drug group**	**Any trimester % (N)**	1^st^ Trimester % (N)	2^nd^ Trimester % (N)	3^rd^ Trimester % (N)
**Antibacterial**	**37% (260)**	13% (90)	16% (115)	18% (129)
*UTIantibiotic*	**21% (151)**	7% (46)	9% (67)	11% (75)
*Pivmecillinam*	**19% (131)**	5% (36)	8% (56)	9% (66)
*Sulfamethizole*	**4% (25)**	2% (13)	1% (8)	1% (6)
*Nitrofurantoin*	**1% (10)**	0% (2)	1% (5)	1% (5)
*RTIantibiotic*	**21% (145)**	7% (47)	8% (55)	9% (65)
*Penicillin*	**15% (107)**	5% (36)	6% (42)	6% (39)
*Ampicillin Derivate*	**6% (41)**	2% (12)	2% (14)	3% (20)
*Macrolide*	**2% (16)**	1% (4)	0% (3)	1% (9)
*Otherantibiotic*	**1% (10)**	1% (4)	0% (2)	1% (4)
*Dicloxacillin*	**1% (5)**	0% (2)	0% (2)	0% (1)
*Metronidazole*	**1% (4)**	0% (1)	0% (0)	0% (3)
*Tetracycline*	**0% (1)**	0% (1)	0% (0)	0% (0)

### Predictors of Antibiotic Use

#### Treatments with UTI antibiotics

In the univariate tests, we found treatment with UTI antibiotics significantly associated with maternal age, maternal smoking, and household income. After covariate adjustment, only maternal age; adjusted odds ratio (aOR) 0.94, 95% CI, [0.90-0.98], *p* = 0.003, and maternal smoking; aOR 1.97, 95% CI, [1.07-3.63], *p* = 0.030 remained significantly associated with the prevalence of UTI antibiotic usage. All other covariates were removed in the multivariate backward selection process. We observed no significant influence from ethnicity, asthma status, alcohol intake, parity, older children in the home, or educational level. (Table 2) 

**Table 2:** Demographic characteristics for the entire cohort and grouped according to administration of UTI antibiotics and RTI antibiotics during pregnancy.

	All	UTI antibiotic		p-value	RTI antibiotic		p-value
		**YES**	**NO**		**YES**	**NO**	
**All % (N)**	**100% (706)**	**21% (151)**	**79% (555)**	**-**	**21% (145)**	**79% (561)**	**-**
Caucasian % (N)	**96% (671)**	97% (144)	95% (527)	0.530	96% (137)	96% (534)	0.956
Maternal age at birth, mean (SD), years	**32.3 (4.4)**	31.2 (4.3)	32.6 (4.4)	**<0.001**	32.7 (5.0)	32.2 (4.2)	0.204
Asthma history % (N)*	**26% (185)**	24% (36)	27% (149)	0.443	37% (53)	24% (132)	**0.001**
Smoking % (N)	**8% (55)**	13% (19)	6% (36)	**0.012**	8% (12)	8% (43)	0.811
Alcohol > 1 unit / week % (N)	**5% (35)**	7% (10)	5% (25)	0.279	3% (5)	5% (30)	0.346
Previous childbirth % (N)	**54% (382)**	52% (79)	55% (303)	0. 619	66% (95)	51% (287)	**0.002**
Older children, mean (SD), number	**0.8 (0.8)**	0.7 (0.8)	0.8 (0.9)	0.449	0.9 (0.9)	0.7 (0.8)	**0.024**
Maternal educational level				0.055			**0.025**
Low**	**11% (75)**	16% (23)	10% (52)		16% (23)	9% (52)	
Medium**	**58% (406)**	59% (87)	58% (316)		60% (84)	58% (319)	
High**	**31% (214)**	25% (37)	32% (175)		24% (34)	32% (178)	
Household annual income				**0.016**			0.186
Low***	**11% (75)**	17% (25)	9% (50)		9% (12)	12% (63)	
Medium***	**51% (355)**	51% (75)	52% (280)		58% (82)	50% (271)	
High***	**38% (261)**	32% (48)	39% (213)		33% (47)	39% (211)	

Univariate associations are analyzed using chi-square test, student’s t-test, or Wilcoxon rank-sum test.

^*^ : History of doctor diagnosed asthma.

^**^ : Low (elementary school or college graduate), Medium (tradesman or medium length), High (university candidate).

^***^ : Low (below 50.000 Euro), Medium (50.000 - 110.000 Euro), High (above 110.000 Euro).

#### Treatments with RTI antibiotics

In the univariate tests, we found treatment with RTI antibiotics significantly associated with maternal asthma, maternal educational level, previous childbirth and older children in the home. After covariate adjustment, maternal asthma; aOR 1.99, 95% CI, [1.33-2.98], *p* < 0.001, maternal educational level; (low vs. high) aOR 2.32, 95% CI, [1.24-4.35], *p* = 0.011 and previous childbirth; aOR 1.80, 95% CI, [1.21-2.66], *p* = 0.004 all remained significantly associated with the prevalence of RTI antibiotic usage. All other covariates except for maternal age were removed in the multivariate backward selection process. We observed no significant influence with respect to ethnicity, maternal age, smoking, alcohol intake, older children in the home, or household annual income. (Table 2) 

#### Trend analyses for socioeconomic factors

Further analyses on the associations between the socioeconomic factors; maternal educational level and household annual income and oral antibiotic administration during pregnancy were performed by univariate Cochran-Armitage Trend Test. We found significant associations between maternal educational level and prevalence of multiple treatments (low 24%, medium 12%, high 11%; p= 0.019), a consumption of any type of antibiotic (low 47%, medium 39%, high 30%; p= 0.004), UTI antibiotics (low 31%, medium 22%, high 17%; p= 0.021), and RTI antibiotics (low 31%, medium 21%, high 16%; p= 0.009). Furthermore, we found a significant association between household income and consumption of UTI antibiotics (low 33%, medium 21%, high 18%; p= 0.009). Household income was not significantly associated with consumption of any type of antibiotic, RTI antibiotics or multiple treatments during pregnancy. (Table 3) 

**Table 3:** Cochran-Armitage trend test for socio-economic variables; maternal educational level and household annual income.

	Low	Medium	High	p-value
Maternal educational level*				
Multiple treatment***	24% (18)	12% (48)	11% (23)	**0.019**
Any antibiotic	47% (35)	39% (156)	30% (63)	**0.004**
UTI antibiotic	31% (23)	22% (87)	17% (37)	**0.021**
RTI antibiotic	31% (23)	21% (84)	16% (34)	**0.009**
Household annual income**				
Multiple treatment***	16% (12)	14% (49)	10% (27)	0.133
Any antibiotic	44% (33)	38% (134)	33% (86)	0.075
UTI antibiotic	33% (25)	21% (75)	18% (46)	**0.009**
RTI antibiotic	16% (12)	23% (82)	18% (47)	0.701

Effects on number of treatments, and use of any antibiotics, UTI antibiotics and RTI antibiotics in pregnancy.

^*^ : Low (elementary school or college graduate), Medium (tradesman or medium length), High (university candidate).

^**^ : Low (below 50.000 Euro), Medium (50.000 - 110.000 Euro), High (above 110.000 Euro).

^***^ : Received more than one antibiotic treatment during pregnancy.

#### Intrapartum antibiotics

Women treated with intrapartum antibiotics were stratified by caesarian section, as all women giving birth by caesarian section were treated intrapartum antibiotics. In the univariate tests, among women giving birth vaginally, we found intrapartum antibiotic treatment significantly associated with gestational age, maternal smoking, and nulliparity. After covariate adjustment, gestational age; aOR 0.72, 95% CI, [0.61-0.85], *p* < 0.001, maternal smoking; aOR 2.84, 95% CI, [1.33-6.06], *p* = 0.007, and nulliparity; aOR 1.79, 95% CI, [1.06-3.02], *p* = 0.030 all remained significantly associated with administration of intrapartum antibiotics. All other covariates were removed in the multivariate backward selection process. We observed no significant influence on intrapartum antibiotic administration with respect to ethnicity, maternal age, asthma status, alcohol intake, maternal educational level, or household annual income. (Table 4)

**Table 4:** Baseline characteristics for the entire cohort (N=696) and for the stratified group of women giving birth vaginally (N=545).

	All	Intrapartum antibiotics		p-value
		**YES**	**NO**	
**All**	**704**	**33% (229)**	**67% (475)**	**-**
Caesarian section % (N)	**22% (157)**	100% (157)	0% (0)	**<0.001**
**Stratified (no caesarian section)**	**545**	**13% (72)**	**87% (475)**	**-**
Gestational Age, mean (SD), weeks	**40.1 (1.5)**	39.2 (2.1)	40.2 (1.3)	**<0.001**
Caucasian % (N)	**95% (522)**	92% (66)	96% (456)	0.101
Maternal age at birth, mean (SD), years	**32.1 (4.3)**	31.8 (3.9)	32.1 (4.3)	0.539
Asthma history % (N)*	**24% (133)**	24% (17)	24% (116)	0.874
Smoking % (N)	**8% (44)**	18% (13)	7% (31)	**<0.001**
Alcohol > 1 unit / week % (N)	**5% (25)**	8% (6)	4% (19)	0.101
Nulliparity % (N)	**44% (240)**	60% (43)	41% (197)	**0.004**
Maternal educational level				0.879
Low**	**11% (59)**	10% (7)	11% (52)	
Medium**	**57% (308)**	60% (43)	57% (265)	
High**	**32% (172)**	31% (22)	32% (150)	
Household annual income				0.988
Low***	**11% (60)**	11% (8)	11% (52)	
Medium***	**51% (272)**	50% (36)	51% (236)	
High***	**38% (204)**	39% (28)	38% (176)	

Women are grouped according to administration of intrapartum antibiotics. Univariate associations are analyzed using chi-square test, student’s t-test, or Wilcoxon rank-sum test.

^*^ : History of doctor diagnosed asthma.

^**^ : Low (elementary school or college graduate), Medium (tradesman or medium length), High (university candidate).

^***^ : Low (below 50.000 Euro), Medium (50.000 - 110.000 Euro), High (above 110.000 Euro).

## Discussion

### Principal Findings

More than one-third of the women were prescribed oral antibiotics during pregnancy. Overall prevalence of antibiotic use during pregnancy were higher with lower maternal educational level. Usage of UTI antibiotics was furthermore associated with maternal age and maternal smoking, and usage of RTI antibiotics was associated with maternal asthma and previous childbirth. Intrapartum antibiotics were always administered during birth by caesarian section. Usage of intrapartum antibiotics in women giving birth vaginally was associated with gestational age, maternal smoking and nulliparity. 

### Strength and Limitations

The main strength of this study is the well-controlled and implemented designed COPSAC_2010_ pregnancy and birth cohort, which is a replication and extension of the COPSAC_2000_ cohort design. The data analyzed was obtained from 706 pregnant women recruited and closely monitored at the COPSAC clinical research center from 24^th^ gestational week. All information on pregnancy exposures and socioeconomic background was collected longitudinally at the visit following the event of interest by a dedicated team, trained according to standard operation procedures, which increases the consistency of the collected data. 

The high reliability of the data obtained on maternal administration of oral antibiotics is a further strength of this study. This information was obtained through interviews with the participants during their visit to the COPSAC clinic and validated against recordings from The Danish Central Medical Register. This is a highly accurate register for data validation as antibiotics may only be prescribed by an authorized physician and can only be purchased from authorized pharmacies. 

It is a limitation to the study that we only have self-reported data on the administration of intrapartum antibiotics. However, this data was obtained by personal interview one week postpartum with the possibility to examine the birth journal.

Another limitation to the study is our lack of information of the time and type of antibiotics administered during childbirth. This represents a limitation to the depth of the data analyses, but did not influence on the study plan. 

Finally, it is not possible to exclude that other lifestyle-related factors, than those available to us may confound our results. 

### Interpretation

We observed a high prevalence (37%) of oral antibiotic usage in pregnancy compared to previous Danish studies (29%)[2,5]. This is quite substantial in a country like Denmark with low consumption of antibiotics in general, however we often observed treatments not recalled by the woman until the register validation, a factor that may lead to underreporting in other studies. The high prevalence of antibiotic administration in our study match the records from the Register of Medicinal Product Statistics, which describe a general increased usage of antibiotics in Denmark[20]. This could represent either an increased rate of infections and/or a lower threshold for treatment than before. 14% of the pregnant women received more than one antibiotic treatment throughout pregnancy, maybe caused by a propensity to recurrent UTIs found in certain women[21] or by some of the predictors found in our analyses. The lowest usage of antibiotics was observed in the first trimester of pregnancy and may be explained by a general principle to avoid drug exposure during the first trimester when fetal organogenesis takes place[9]. The higher prevalence observed in the third trimester could be explained by the physiologic changes associated with the fetal growth through pregnancy[22,23].

About half of pregnant women will experience symptoms of UTI during pregnancy, but these symptoms are rarely attributed to bacteriuria[24,25]. Nevertheless treatment of asymptomatic bacteriuria is recommended in pregnancy, as it can lead to pyelonephritis due to pregnancy-induced dilatation of the urinary tract and reflux, and represents a risk of severe morbidity to mother and child[22]. Dipsticks positive for nitrites and leukocyte esterase are considered insufficient to initiate antibiotic treatment; however many women are treated without further culturing[26,27]. Thus, some women may have been treated unnecessarily and risk of antibiotic resistance is to be considered. 

We observed an intrapartum antibiotic prevalence of 33% in the COPSAC_2010_ pregnancy cohort, most often associated with caesarian section and 13% among the women giving birth vaginally. Intrapartum antibiotic prophylaxis is recommended for delivery by caesarian section, as it reduces postpartum infections in the mother[14]. The screening procedure for GBS is not implemented in Denmark. Instead, administration of prophylactic intrapartum antibiotics is based on a risk factor approach, which requires intrapartum maternal fever >38°C, rupture of membranes >18 h to be present or women presenting with true preterm labor (<37 weeks gestation)[11–13]. 

In Denmark, all citizens have free and equal access to health care services. This makes Denmark suitable for conducting analyses on predictors for antibiotic administration. Furthermore, only a limited number of antibiotic types are used in pregnancy. By grouping the antibiotic treatments by suspected indication, we were able to differentiate between two individual outcomes. Thereby, we hope for a better understanding of the mechanisms behind the observations. We have shown an important association between oral antibiotic usage and maternal educational level. The prevalence of multiple treatments as well as treatment with both UTI antibiotic and RTI antibiotic among the lower educated were almost twice the prevalence of the higher educated. This matches another study describing increased antibiotic usage among women who were welfare recipients, unemployed or pensioners[3]. UTI antibiotics was furthermore associated with maternal age and smoking, and remained significant after adjustment for covariates. We expect these observations to be lifestyle-related and may be a result of a lower threshold for attending the general practitioner. Previous antibiotic administration in pregnancy and frequent visits to the physician has been associated with a higher risk of subsequent treatment[4]. Asthmatic mothers showed higher prevalence of RTI antibiotic usage. Asthmatics tend to have more RTIs than non-asthmatics and these may trigger asthmatic episodes in susceptible individuals[28]. We suspect the threshold for initiating antibiotic treatment to be lower in asthmatics, who might be further susceptible to infection because of pregnancy[23,29]. Previous childbirth was associated with use of RTI antibiotics in pregnancy, which could be explained by transmission of airway pathogens from children in the home. 

After stratification for vaginal birth and adjusting for covariates, we found a higher prevalence of intrapartum antibiotic usage associated with a lower gestational age, nulliparity, and a treatment incidence almost three-fold higher among mothers who smoked during pregnancy. We found no association with either maternal educational level or household income; hence the association with intrapartum antibiotic usage observed is believed to be a result of the individual factors rather than the socio-economic background. The association with nulliparity, is suggested to be a result of prolonged labor, more often seen in women giving birth for the first time[30]. Smoking is generally recognized as contributing factor to an overall increased risk to health. In pregnancy, it is linked to reduced fetal growth and placental development[31–33], and has also been associated with an increased risk of prolonged postpartum bleeding and perioperative complications including infections[34,35].

Our findings suggest an association between maternal social and educational status and intake of antibiotics during pregnancy. This may represent a general lower health status among these women, but perhaps more likely, it may be caused by treatment-seeking behavior of the woman[3]. These risk factors have to be investigated further to evaluate causality. 

## Conclusions

The prevalence of oral antibiotic administration during pregnancy found in our study match the general increase in consumption observed in Denmark over the last decade with more than one-third of pregnant women being treated. Any oral antibiotic treatment as well as use of more than one treatment during pregnancy was associated with lower maternal educational level. Lower maternal age and smoking was associated with usage of UTI antibiotics and asthmatic women and women with previous childbirth had a higher prevalence of RTI antibiotic use. Administration prevalence of intrapartum antibiotics was higher in smokers, in women giving birth at a lower gestational age, and in women without previous childbirth. These predictors for antibiotic administration may be used as general health indicators, and may guide preventive strategies in order to avoid unnecessary use of antibiotics.
